# Peptide bonds strike back

**DOI:** 10.1107/S2052252525003604

**Published:** 2025-04-25

**Authors:** Ashwin Chari

**Affiliations:** a Research Group for Structural Biochemistry and Mechanisms Max Planck Institute for Multidisciplinary Sciences Am Fassberg 11 D-37077Göttingen Germany

**Keywords:** peptide bonds, protein structure prediction, protein design, keto–enol tautomerism

## Abstract

Recent advances in protein design and protein structure prediction have questioned whether experimental structural biology still has a role to play in research today. The article by Panjikar and Weiss [(2025). *IUCrJ*, **12**, 307–321] partially answers this question and alludes to a role still to be played by structural biology. Several properties of peptide bonds, likely important for function, are described that are absent in protein design and predicted protein structures, and that have largely been overlooked by the structural biology community.

Protein structure prediction and design has profoundly changed biological research in general and structural biology in particular. The significance of advances in these two areas in recent years is highlighted by the fact that the Nobel Prize in Chemistry in 2024 was awarded to David Baker for computational protein design and jointly to Demis Hassabis and John Jumper, who developed machine-learning-based *AlphaFold* protein structure prediction (Jumper *et al.*, 2021 ▸). It was William DeGrado, Stephen Mayo and David Baker who pioneered the computational design of proteins (Dahiyat & Mayo, 1997 ▸; Regan & DeGrado, 1988 ▸), with David Baker and his team being the first to design a protein with a completely new fold (Kuhlman *et al.*, 2003 ▸). Protein structure prediction and protein design similarly were heavily dependent on experimentally determined structural information in the Protein Data Bank (PDB; Berman *et al.*, 2000 ▸). However, today the number of predicted protein structures exceeds the number of experimentally determined structures almost tenfold, and protein design is able to generate myriads of proteins *de novo*, almost at will (Buller *et al.*, 2025 ▸; Listov *et al.*, 2024 ▸). This questions if and how experimental structural biology has a role to play in research nowadays?

The article *Peptide bonds revisited* in the current issue of *IUCrJ* (Panjikar & Weiss, 2025 ▸) partially addresses this question, and highlights the importance still to be played by experimental structural biology, especially high-resolution protein crystallography. The authors expand the analysis of Weiss and colleagues (Weiss *et al.*, 1998 ▸), and observations of deviations from commonly assumed peptide bond geometry made at high resolution (Hendrickson & Teeter, 1981 ▸; Podjarny *et al.*, 2003 ▸; Dauter *et al.*, 1995 ▸). Panjikar and Weiss compile a non-redundant set of 1024 high-resolution protein structures (better than 1.2 Å) from the PDB. They utilize this set of protein structures to examine the distinct characteristics of peptide bonds in α-helices and β-strands. Their analysis reveals surprising and intriguing insights into bond lengths, angles, dihedral angles, electron density distributions and hydrogen bonding in structural elements. While no specific difference in bond lengths is found between the two secondary structure elements, the bond angles in β-strands are significantly larger than in α-helices. Similarly, the distribution of dihedral angles in β-strands spans a larger range than in α-helices. Both angle distributions allude to specific conformational preferences of the respective secondary structure motifs. A skewed distribution of absolute electron density is observed, with a lower ratio of C=O to C—N bonds in helices than for strands and higher normalized atomic displacement parameters (ADPs) for peptide atoms in helices relative to strands. Panjikar and Weiss suggest that α-helical peptide bonds seem to display more enol-like character than peptide bonds in β-strands, which is often accompanied by positive difference density alluding to protonation of the C—O bond (Fig. 1 ▸).

The observations made by Panjikar & Weiss point to several properties of peptide bonds which are absent from any *AlphaFold* or *RosettaFold* prediction but are most likely important for protein function in general. Beyond protein structure prediction and protein design, their findings are certainly astute with regards to local restraint definitions in protein structure refinement, and point to dynamics of peptide bonds that have largely been overlooked by the structural biology community. There are compelling implications for refining protein structures by applying more flexible restraints in α-helical regions where enol-like transitions of peptide bonds may arise. This could improve the accuracy of modelled local geometries and aid efforts such as *PDB-REDO* (Joosten *et al.*, 2012 ▸, 2009 ▸), leading to improved protein models serving as templates for protein structure prediction and protein design.

## Figures and Tables

**Figure 1:**
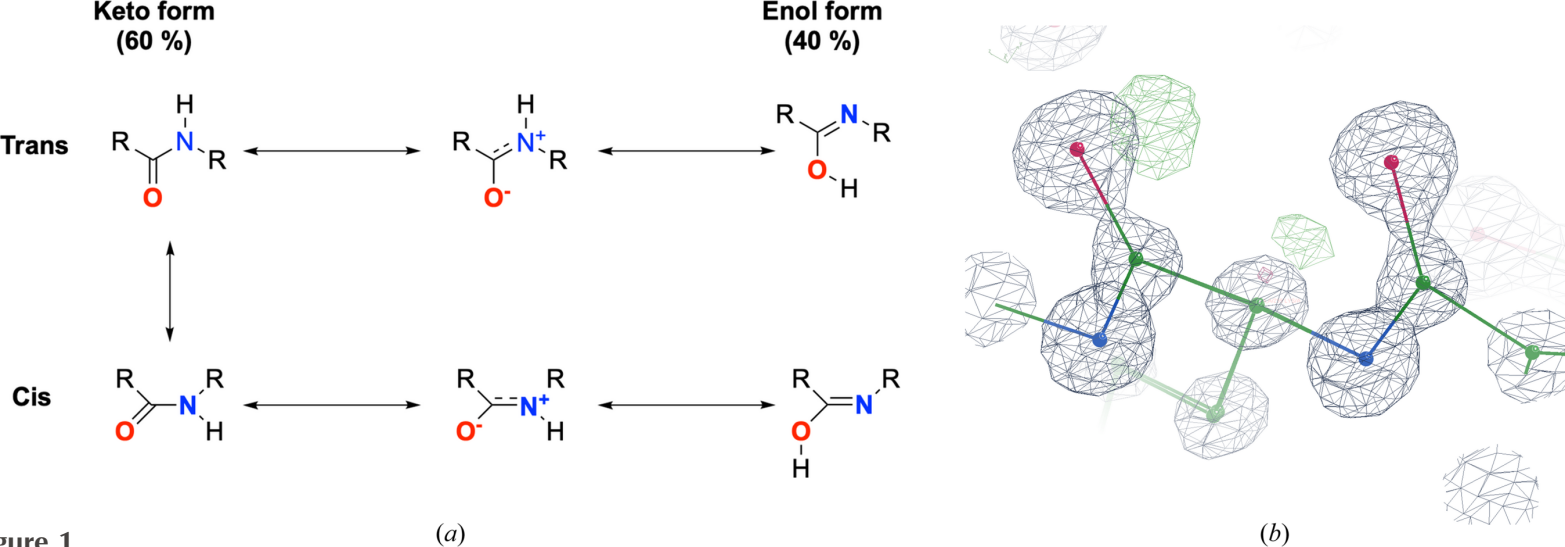
(*a*) Chemical drawings representing keto–enol tautomerism in *cis*- (lower row) and *trans*- (upper row) peptide bonds. Prepared with *ChemDrawTM* (Revvity Signals Software, Inc.). (*b*) Keto–enol tautomerization in PDB entry 6mu9. Shown are two peptide bonds between amino acids 298–300. Note the hydrogen-omit signal on the left peptide carbonyl, indicating that the C—O bond is protonated. 2*mF*_o_− *F*_c_ (grey) and *mF*_o_ − *F*_c_ (green, positive) densities are both contoured at 3rms. Prepared with *Coot* version 0.98.96 (Emsley & Cowtan, 2004 ▸).
